# The *FADS1* rs174550 Genotype Modifies the n‐3 and n‐6 PUFA and Lipid Mediator Responses to a High Alpha‐Linolenic Acid and High Linoleic Acid Diets

**DOI:** 10.1002/mnfr.202200351

**Published:** 2022-11-11

**Authors:** Topi Meuronen, Maria A. Lankinen, Johan Kolmert, Vanessa Derenji de Mello, Taisa Sallinen, Jyrki Ågren, Kirsi A. Virtanen, Markku Laakso, Craig E. Wheelock, Jussi Pihlajamäki, Ursula Schwab

**Affiliations:** ^1^ Institute of Public Health and Clinical Nutrition School of Medicine University of Eastern Finland Kuopio 70211 Finland; ^2^ Food Sciences Unit University of Turku Turku 20500 Finland; ^3^ Unit of Integrative Metabolomics Institute of Environmental Medicine Karolinska Institutet Stockholm 171 65 Sweden; ^4^ University of Eastern Finland Library Kuopio Kuopio 70600 Finland; ^5^ Institute of Biomedicine School of Medicine University of Eastern Finland Kuopio 70211 Finland; ^6^ Department of Medicine Endocrinology and Clinical Nutrition Kuopio University Hospital Kuopio 70210 Finland; ^7^ Institute of Clinical Medicine Internal Medicine University of Eastern Finland Kuopio 70029 Finland; ^8^ Department of Medicine, Kuopio University Hospital Kuopio 70210 Finland; ^9^ Department of Respiratory Medicine and Allergy Karolinska University Hospital Stockholm 141 86 Sweden; ^10^ Gunma University Initiative for Advanced Research (GIAR) Gunma University Maebashi 371‐8511 Japan

**Keywords:** alpha‐linolenic acid, octadecanoid, eicosanoid, eicosapentaenoic acid, FADS, linoleic acid

## Abstract

**Scope:**

The fatty acid composition of plasma lipids, which is associated with biomarkers and risk of non‐communicable diseases, is regulated by dietary polyunsaturated fatty acids (PUFAs) and variants of fatty acid desaturase (*FADS*). We investigated the interactions between dietary PUFAs and *FADS1* rs174550 variant.

**Methods and results:**

Participants (*n* = 118), homozygous for *FADS1* rs174550 variant (TT and CC) followed a high alpha‐linolenic acid (ALA, 5 percent of energy (E‐%)) or a high linoleic acid (LA, 10 E‐%) diet during an 8‐week randomized controlled intervention. Fatty acid composition of plasma lipids and PUFA‐derived lipid mediators were quantified by gas and liquid chromatography mass spectrometry, respectively. The high‐LA diet increased the concentration of plasma LA, but not its lipid mediators. The concentration of plasma arachidonic acid decreased in carriers of CC and remained unchanged in the TT genotype. The high‐ALA diet increased the concentration of plasma ALA and its cytochrome P450‐derived epoxides and dihydroxys, and cyclooxygenase‐derived monohydroxys. Concentrations of plasma eicosapentaenoic acid and its mono‐ and dihydroxys increased only in TT genotype carriers.

**Conclusions:**

These findings suggest the potential for genotype‐based recommendations for PUFA consumption, resulting in modulation of bioactive lipid mediators which can exert beneficial effects in maintaining health.

## Graphical abstract

This study aimed to explore the differences in the plasma fatty acid and lipid mediator concentrations in response to dietary polyunsaturated fatty acids (PUFA) between carriers of the *FADS1* rs174550 TT and CC genotypes. Plasma concentrations of long‐chain PUFAs and their derived bioactive lipid mediators changed in a genotype dependent manner in response to linoleic and alpha‐linolenic acid enriched diets.

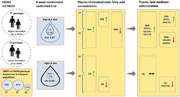

## 1 Introduction

**Figure 1:**
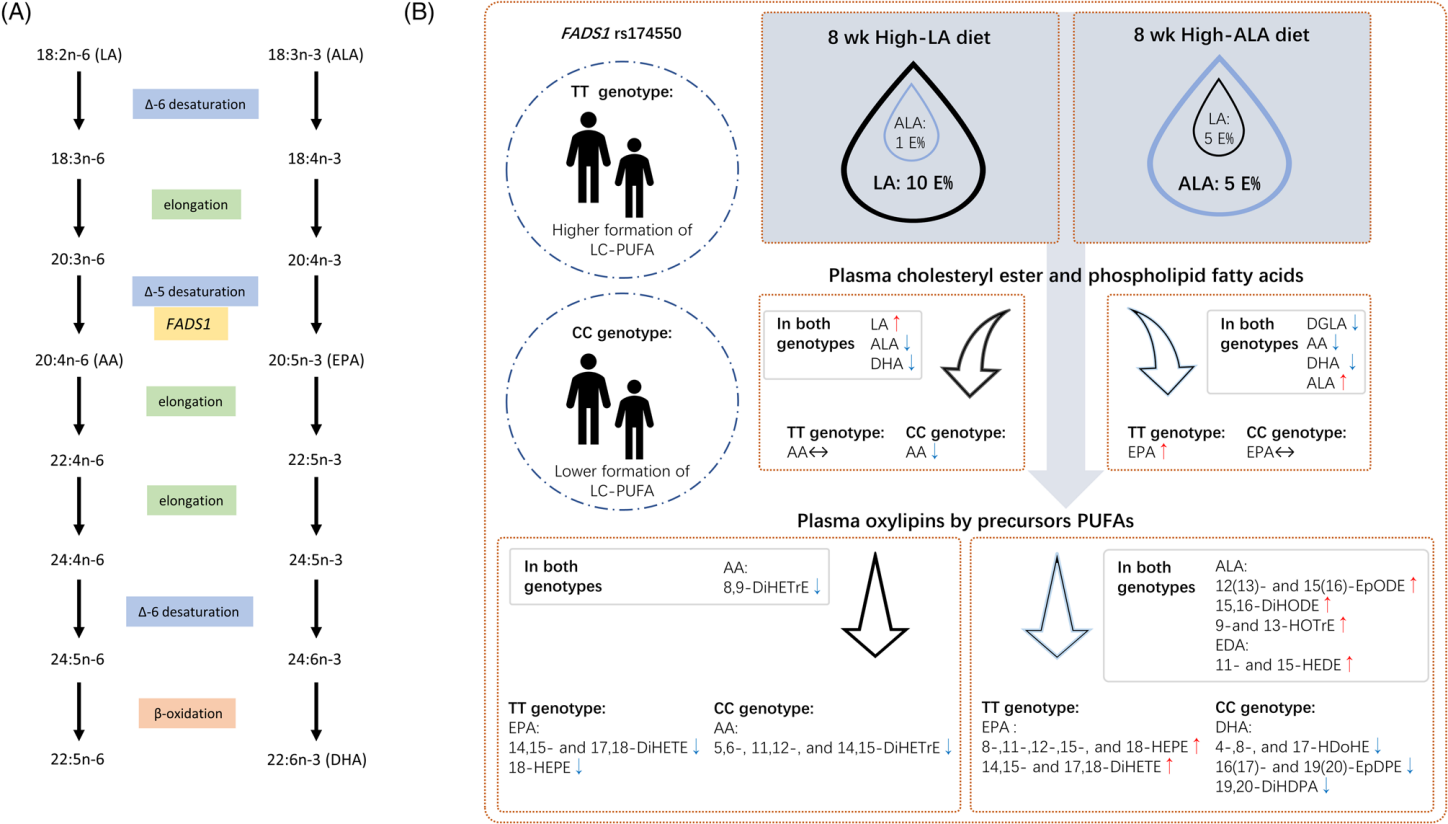
A) Metabolic pathway of n‐6 and n‐3 PUFAs. B) Study design and the main results of the study. Participants with *FADS1* rs174550 TT and CC genotypes were randomized to receive either high‐LA or high‐ALA diet for 8 weeks. Direction of the changes in the concentration of plasma cholesteryl ester and phospholipid PUFAs and lipid mediator concentrations are illustrated with arrows, genotype (TT or CC) specific changes and shared changes in response to diets are separated.

Fatty acid composition of plasma lipids is not solely determined by their dietary intake,^[^ ^1^ ^]^ but is also affected by genetic variation in the fatty acid desaturase (*FADS*) gene cluster. The delta‐5‐desaturase enzyme (D5D), encoded by the *FADS1* gene, is needed for the biosynthesis of the long‐chain PUFAs, such as arachidonic acid (AA) and eicosapentaenoic acid (EPA) from linoleic acid (LA) and α‐linolenic acid (ALA),^[^ ^2^ ^]^ respectively (**Figure** **1**A). *FADS1* variants that are associated with increased gene expression of *FADS1* in the liver^[^ ^3^, ^4^ ^]^ also show higher D5D index^[^ ^5^ ^]^ leading to increased AA:LA and EPA:ALA ratios in plasma^[^ ^6^, ^7^ ^]^ compared to carriers of alleles associated with decreased gene expression of *FADS1*. Variants that are associated with decreased expression of *FADS1* and *FADS2* are therefore associated with higher serum^[^ ^8^ ^]^ and plasma^[^ ^9^, ^10^ ^]^ proportions of LA and ALA and lower AA and EPA, but not with altered docosahexaenoic acid (DHA).

PUFAs are converted via enzymatic processes to downstream lipid mediators that exert potent biological functions. For example, AA serves as substrate to form the eicosanoids, which are well‐known lipid mediators in infection, inflammation, T2D, and cardiovascular diseases.^[^ ^11^, ^12^, ^13^ ^]^ The n‐6 and n‐3 PUFAs can compete as substrates for the enzymes responsible for producing bioactive lipid mediators, which has resulted in an interest in how health status may be influenced by the relative ratios of dietary PUFAs.

Higher proportions of n‐6 LA, but not long‐chain AA, in plasma fatty acids, are associated with decreased incidence of T2D^[^ ^14^ ^]^ and cardiovascular diseases.^[^ ^15^ ^]^ Higher proportions of long‐chain n‐3 PUFAs EPA, docosapentaenoic acid (DPA), and DHA in circulation are associated with lower incidence of T2D; however, ALA is not.^[^ ^16^ ^]^ ALA, DPA, and DHA are associated with lower risk of fatal coronary heart disease.^[^ ^17^ ^]^ Increases in dietary LA, in exchange of carbohydrates or saturated fat, are associated with decreased risk of T2D.^[^ ^18^ ^]^ Results regarding n‐3 PUFAs and their role in development of T2D or frequency of cardiovascular events are inconclusive.^[^ ^18^ ^]^

The ability of humans to efficiently convert LA and ALA to AA and EPA, respectively, is of a great nutritional debate because studies on the conversion of ALA to EPA in humans are contradictory.^[^ ^19^, ^20^ ^]^ Absolute amount of LA and ALA in the diet is likely to affect the conversion of LA and ALA into AA and EPA, respectively.^[^ ^21^ ^]^ In addition to national differences in the dietary PUFA composition also allelic frequencies of SNPs in *FADS* gene cluster varies across geographic regions.^[^ ^22^ ^]^ Dietary PUFAs and *FADS* variants contribute to the differences in concentrations of main circulatory PUFAs, and possibly their downstream metabolite (oxylipins) concentrations. Therefore, targeted analyses of both primary plasma fatty acids and their derived lipid mediators, as response to dietary challenges, can improve our understanding of mechanisms behind health effects of dietary PUFAs, in relation to gene variants.

A common intronic variant *FADS1* rs174550 strongly regulates plasma lipid PUFA composition and associates with fasting plasma glucose, triglyceride, and HDL‐C concentrations.^[^ ^3^, ^23^, ^24^ ^]^ We aimed to study how the genetic variant *FADS1* rs174550 (homozygote) interacts with dietary intakes of LA and ALA during an 8‐week intervention and analyzed changes in plasma fatty acid composition and lipid mediators.

## 2 Experimental Section

### 2.1 Subjects and Interventions

**Figure 2:**
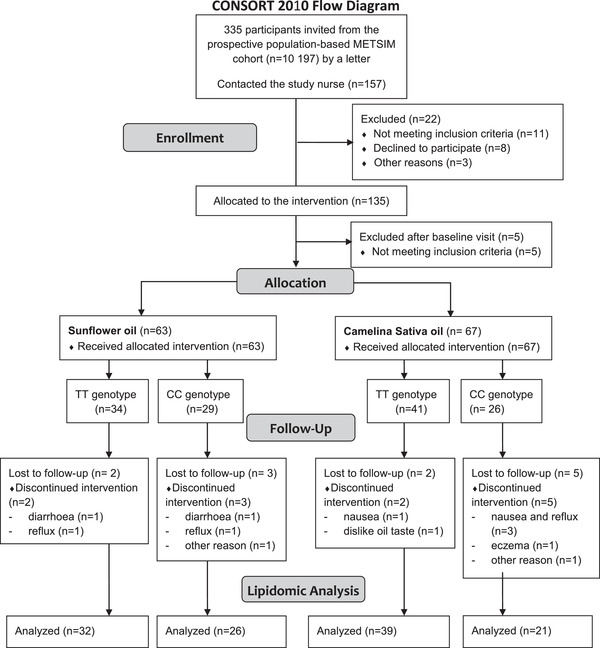
Consort flow diagram.

Male Caucasian participants homozygous for *FADS1* rs174550 SNP, *n* = 71 and *n* = 47, for TT and CC genotypes respectively, were recruited from the Metabolic Syndrome in Men (METSIM) study (Consort flow diagram, **Figure** **2**). Clinical baseline characteristics by genotype showed no significant differences on age (years) 66.6 ± 5.6 (TT) and 64.8 ± 5.6 (CC), BMI (kg m^−2^) 24.6 ± 2.6 (TT) and 24.8 ± 2.6 (CC), fasting plasma glucose (mmol L^−1^) 5.8 ± 0.4 (TT) and 5.8 ± 0.5 (CC).^[^ ^25^ ^]^ A *FADS1* variant rs174550 was genotyped using the TaqMan SNP Genotyping Assay (Applied Biosystems, Foster City, CA, USA) according to their protocol. A full description of the study design was reported elsewhere.^[^ ^25^ ^]^ In brief, after a 4‐week run‐in period, participants were randomized based upon their *FADS1* rs174550 genotype, BMI, age, and fasting plasma glucose concentration to receive diet either rich in LA or ALA (Figure 1B and  2). Subjects were instructed to consume sunflower oil (SFO) (high‐LA diet) or *Camelina sativa* oil (CSO) (high‐ALA diet) daily during an 8‐week intervention period. Fatty acid composition of the oils is reported in supplementary materials (Table S1, Supporting Information). Supplemented dose of oil was depending on participants’ baseline BMI (BMI 20–24 kg m^−2^ 30 mL, BMI 24.1–28 kg m^−2^ 40 mL, and for BMI 28.1–32 kg m^−2^ 50 mL). Oil was consumed unheated. Participants were instructed to consume meat and dairy products with low fat content to keep total dietary fat intake constant during the intervention. Four‐day food records were collected once in the run‐in period and twice (at week 4 and 8) during the intervention and daily consumption records of oil were collected. Compliance to oil consumption and study diets were estimated by the consumption records and food records by a clinical nutritionist. The mean consumption of oils was 29.7 mL day^−1^ for those who aimed to consume 30, 39.1 mL day^−1^ for those who aimed to consume 40 and 46.9 mL day^−1^ for those who aimed to consume 50 mL day^−1^, as reported in Lankinen et al.^[^ ^25^ ^]^ Fasting plasma samples, after >10 h fasting, were collected at the baseline and after 8‐week intervention. All samples from participants who finalized the intervention were analyzed. The sample size was calculated based on the high‐LA diet induced changes in the proportions of plasma cholesteryl ester AA between the TT and CC genotypes (4.20 and 12.14 mol%, respectively) in the FADSDIET1 trial,^[^ ^5^ ^]^ with the *β* = 0.8 and *α* = 0.025, instead of only 0.05, to account for the fact that the study had two dietary interventions instead of one. Therefore, assuming a 15% dropout rate, 35 participants in each study group was needed.^[^ ^25^ ^]^ This study was conducted according to the guidelines laid down in the Declaration of Helsinki. The study plan was approved by the Ethical committee of the Hospital District of Northern Savo (516/2018) and the study was registered with clinicaltrials.gov (NCT03572205). Written informed consent was obtained from all participants.

### 2.2 Fatty Acid Measurements

The fatty acid composition of plasma lipids in four fractions, cholesteryl esters (CE), phospholipids (PL), triglycerides (TG), and free fatty acids (FFA) were quantified as previously described.^[^ ^26^, ^27^ ^]^ As suggested by authors^[^ ^28^ ^]^ the total concentrations of individual fatty acids as a main outcome in the analyses independently of the concentrations of other fatty acids were used. In addition, relative proportions of each fatty acid (mol% data) calculated as percentage of total measured fatty acid in each fraction were used.

D5D index was estimated as the AA/dihomo‐γ‐linolenic acid (DGLA) ratio and D6D index as γ‐linolenic acid (GLA)/LA. Index of aggregated desaturase activity (ADA) was estimated for n‐6 and n‐3 PUFAs as AA/LA and EPA/ALA, respectively. Indices of elongation of very long chain fatty acid (ELOVL) enzyme, were estimated as a ratio of adrenic acid /AA, DPA/EPA, and DGLA/GLA.

### 2.3 Lipid Mediator Profiling

Lipid mediator quantitation was performed by liquid chromatography–mass spectrometry, as previously described.^[^ ^29^, ^30^ ^]^ In addition, the ALA‐derived epoxide, 15(16)‐epoxyoctadecadienoic acid (EpODE) was semi‐quantified using the 12(13)‐EpODE standard curve assuming similar ESI response factor.

### 2.4 Statistical Analyses

Lipid mediators, desaturase and elongase indices, and fatty acids (mol% and concentration data separately) in each lipid fractions were analyzed in individual batches. Data were normalized by inverse normal transformation. Baseline differences between *FADS1* rs174550 TT and CC genotypes were compared using linear model with genotype as an explanatory variable. Differences between genotype responses to each supplemented oil over time were compared using separate linear mixed‐effect (LME) models: genotype + timepoint + genotype × timepoint, with subject identifier as a random effect variable. Within‐genotype response, to supplemented oil, was analyzed by a linear mixed‐effect model: timepoint as an explanatory variable and subject identifier as a random effect variable. FDR correction was applied for *p*‐values individually for each set of measurements. For fatty acids the FDR adjusted *p* < 0.05 were considered statistically significant. Since power calculations were based on fatty acids, also nominally significant (*p*_unadjusted_ < 0.05) results were considered for lipid mediators. R‐packages ggpubr (version 0.4.0) and ComplexHeatmap (version 2.2.0)^[^ ^31^ ^]^ were used for data visualization.

## 3 Results

One‐hundred and eighteen subjects completed the study and 236 plasma samples (0 and 8 weeks) were successfully quantified to report fatty acids in CE, PL, TG, and FFA fractions. Analytical batch‐to‐batch averages of all fatty acids in all four fractions demonstrated a coefficient of variation (CV) of 12.4% and 6.2% for concentration and proportions, respectively. In addition, 71 polyunsaturated fatty acid metabolites (oxylipins) in plasma could be reported, having satisfying quality control precision (average CV of 7.5%).

### 3.1 FADS1 rs174550‐TT Genotype is Associated with High Desaturase Indices and Long‐Chain PUFA Concentrations in Plasma Lipids at Baseline

The plasma PUFA concentrations and proportions in the PL, CE, TG, and FFA fractions are presented in the supplementary materials (Tables S2–S9, Supporting Information). As expected, carriers of TT‐genotype had higher indices of D6D in the CE fraction, D5D in the CE and PL fractions, AA/LA and EPA/ALA ratios in the CE and PL fractions (Figure S1, Supporting Information) at baseline. GLA was not quantified in the PL fraction due to low concentration and therefore D6D was not estimated in the PL fraction. The concentrations of AA in the CE and PL fractions and EPA in the CE and PL (*p*_unadjusted_ = 0.021) fractions were higher in the carriers of the TT genotype compared with carriers of the CC genotype. There were no differences in DHA between the genotypes in any measured fractions (Figures S1–2, Tables S2–5, Supporting Information). The concentrations of LA in the PL fractions and ALA in the CE and PL fractions were higher in the carriers of the CC genotype. Our results suggest that the carriers of the TT and CC genotypes have differences in the plasma n‐6 PUFA derived lipid mediator concentrations. Compared with the carriers of the CC genotype the carriers of the TT genotype had nominally (*p*_unadjusted_ < 0.05) higher concentrations of AA‐derived cytochrome P450 (CYP) metabolites (5,6 and 8,9‐dihydroxyeicosatrienoic acid (DiHETrE)) and DGLA‐derived monohydroxy fatty acids (5‐ and 8‐ hydroxyeicosatrienoic acid (HETrE)) (Table S10, Supporting Information).

### 3.2 Dietary Intakes of Essential FAs Increased in Response to Study Diets

According to food records high‐LA and high‐ALA diets increased dietary intake of LA and ALA, respectively, both in mg and expressed as a percentage of total energy (*E*%) (Table S12, Supporting Information). During the high‐LA diet, the proportion of LA of total energy increased from ≈3 to ≈10 E%. During the high‐ALA diet, the ALA intake increased from ≈1 to ≈5 E% and LA from ≈3 to ≈5 E%. The ratio of LA to ALA increased in response to high‐LA and decreased in response to high‐ALA diet from 4:1 to ≈12:1 and ≈4:1 to ≈1:1, respectively. Dietary intakes of EPA and DHA remained unchanged in all groups. Within high‐LA and high‐ALA study arms dietary intakes were similar among the genotype groups (Table S12, Supporting Information).

### 3.3 High‐LA Diet Decreased AA Concentrations in Plasma Lipids in the Carriers of *FADS1* rs174550‐CC Genotype

**Figure 3:**
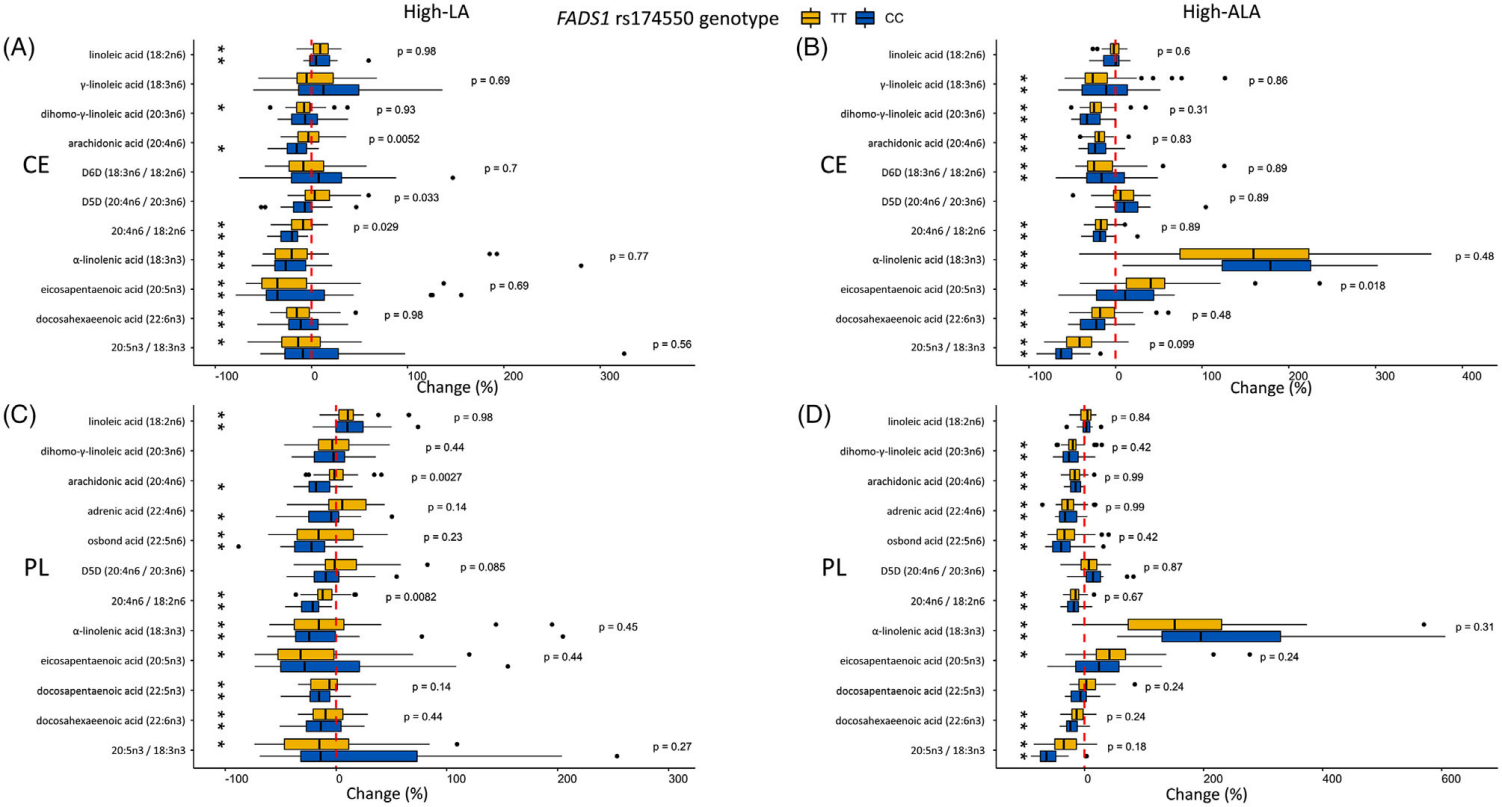
Changes (%) in PUFA concentrations in plasma cholesteryl esters in the A) high‐LA and B) high‐ALA diets and plasma phospholipids in the C) high‐LA and D) high‐ALA diets. The dotted red line is set to 0% in each graph. Black asterisk (*) shows FDR corrected *p*‐values <0.05 within the study group changes (0 wk vs 8 wk) and *p*‐value on the right side the FDR corrected *p*‐values for genotype–diet interaction.

In response to high‐LA diet, the concentrations of LA increased in the PL, CE fractions (**Figure** **3**A,C), and TG fractions (Table S8, Supporting Information). There were genotype‐diet interactions for D5D index and AA in the CE fraction. D5D index remained unchanged in the carriers of the TT genotype but tended to decrease in the carriers of the CC genotype in the CE fraction. The concentration of AA in the CE and PL fractions decreased in the carriers of the CC genotype but remained unchanged in the carriers of the TT genotype. The estimated D6D and concentration of GLA in the CE fraction remained unchanged in response to the high‐LA diet. Concentrations of total MUFAs and ALA, DPA, and DHA decreased in both genotypes in the CE and PL fractions in response to the high‐LA diet (Tables S6,S7, Supporting Information).

### 3.4 A High‐LA Diet does not Increase LA‐ and AA‐Derived Lipid Mediators

**Figure 4:**
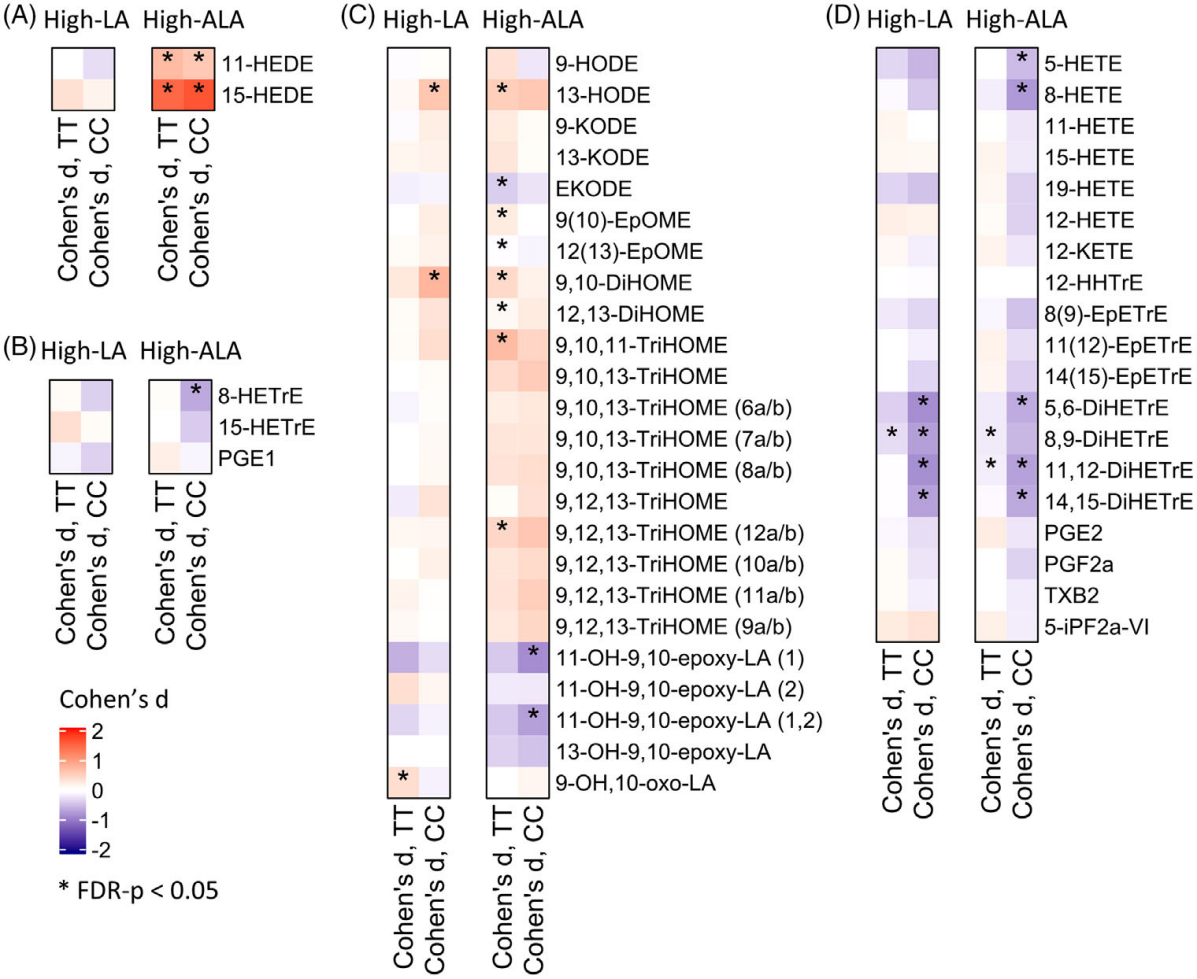
Changes in plasma A) EDA, B) DGLA, C) LA, and D) AA derived lipid mediator concentrations. Values are Cohen *D* values (0 wk vs 8 wk) and red and blue colors indicate increased and decreased concentrations, respectively. Black asterisk (*) shows FDR corrected *p*‐values <0.05 within the study group changes (0 wk vs 8 wk). Structural isomers of 11‐OH‐9,10‐epoxy‐LA are annotated as 1 and 2 and 9,10,13‐ and 9,12,13‐TriHOMEs as in ref. [30].

**Figure 5:**
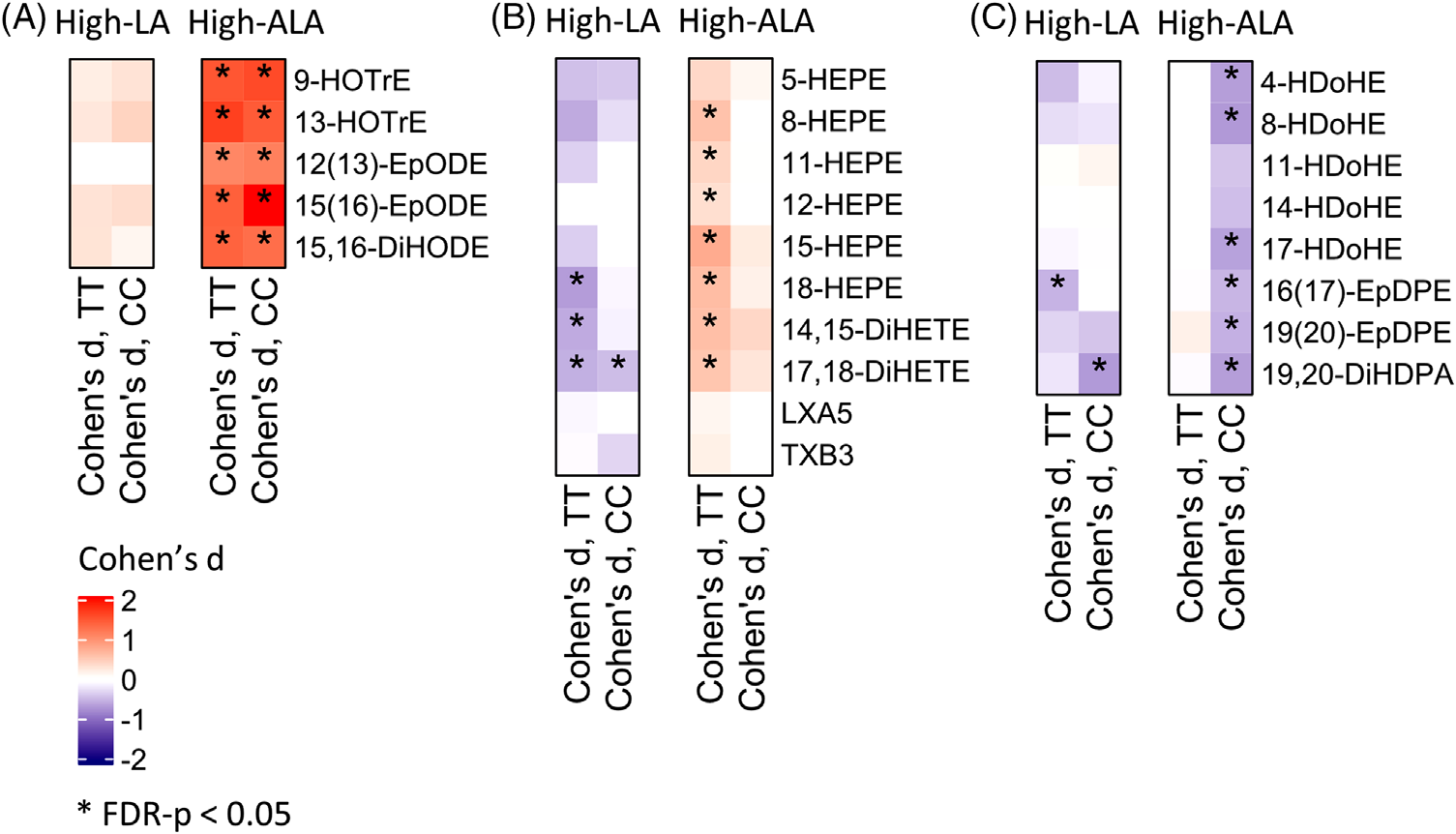
Changes in plasma A) ALA, B) EPA, and C) DHA derived lipid mediator concentrations. Values are Cohen *D* values (0 wk vs 8 wk) and red and blue colors indicate increased and decreased concentrations, respectively. Black asterisk (*) shows FDR corrected *p*‐values < 0.05 within the study group changes (0 wk vs 8 wk).

Following a high‐LA diet there were no general increase in LA‐derived lipid mediators; in the carriers of the CC genotype there was a modest increase in 13‐hydroxyoctadecadienoic acid (13‐HODE), 9,10‐dihydroxyoctadecenoic acid (9,10‐DiHOME), and 12,13‐DiHOME tended to increase (*p*_unadjusted_ = 0.008) (**Figure** **4**B) (Table S11, Supporting Information). In the carriers of the TT genotype the 9‐OH,10‐oxo‐LA increased and the 9,10‐DiHOME showed a small increase (*p*_unadjusted_ = 0.006). However, there were no genotype–diet interactions in LA‐derived lipid mediators. Lipid mediators derived from DGLA remained unchanged (Figure 4B). For AA‐derived lipid mediators, there were nominally (*p*_unadjusted_ < 0.05) significant genotype–diet interactions for CYP + sEH metabolites 11,12‐ and 14,15‐DiHETrE (Figure 4D) which decreased in the carriers of the CC genotype but remained unchanged in the carriers of the TT genotype. In addition, 5,6‐ and 8,9‐DiHETrE decreased in the carriers of the CC genotype and tended to decrease in the carriers of the TT genotype (*p*_unadjusted_ 0.007 and 0.002, respectively). However, the concentrations of 8,9‐, 11,12‐ and 14,15‐epoxyeicosatrienoic acid (EpETrE) remained unchanged in both genotypes. In general, correlations between the absolute changes of plasma PL LA and AA and LA‐ and AA‐derived lipid mediator concentrations were weak in the high‐LA diet (Figure S3A,B, Supporting information). ALA and DHA derived lipid mediators remained mainly unchanged in the carriers of the CC and TT genotype (**Figure** **5**A,C). However, EPA derived 18‐HEPE and 14,15‐ and 17,18‐DiHETE significantly decreased in the carriers of the TT genotype (Figure 5B).

### 3.5 Plasma Lipid EPA Concentration Increased in Response to a High‐ALA in the Carriers of the *FADS1* rs174550‐TT Genotype

In the high‐ALA diet, the plasma concentrations of ALA increased in the CE, PL (Figure 3B,D), TG, and FFA (Tables S8,S9, Supporting Information) fractions in both genotypes. There was a genotype–diet interaction on the EPA concentration. Concentration of plasma EPA in the CE fraction increased in the carriers of TT genotype in response the high‐ALA diet. Similar trend for genotype–diet interaction (*p*_unadjusted_ = 0.018) for EPA concentration and increase in the TT genotype was observed in the PL fraction. EPA concentrations and proportions remained unchanged in the CE and PL fractions, but nominally increased (*p*_unadjusted_ = 0.036 and 0.047 for proportions and concentrations, respectively) in the TG fraction in the carriers of the CC genotype. Within both genotypes’ concentrations of ≥20C n‐6 PUFAs, including DGLA and AA, and DHA in the CE and PL fractions decreased in response to the high‐ALA diet.

### 3.6 A High‐ALA Diet Increased ALA in both Genotypes and EPA‐Derived Lipid Mediators in the Carriers of the *FADS1* rs174550‐TT Genotype

All measured ALA‐derived lipid mediators (12(13)‐EpODE, 15(16)‐EpODE, 9‐ and 13‐hydroxyoctadecatrienoic acid (HOTrE), and 15,16‐dihydroxyoctadecadienoic acid (DiHODE)) (Figure 5A) and eicosadienoic acid derived 11‐ and 15‐hydroxyeicosadienoic acid (HEDE) (Figure 5A) increased in both genotypes in response to high‐ALA diet. Nominally significant differences were observed between TT and CC genotypes in EPA‐ and DHA‐derived lipid mediators. EPA‐derived lipoxygenase (LOX) metabolites 15‐hydroxyeicosapentaenoic acid (HEPE) (*p*_unadjusted_ = 0.0203 for genotype–diet interaction), 8‐HEPE, 5‐HEPE, and CYP metabolites 14,15‐dihydroxyeicosatetraenoic acid (DiHETE), 17,18‐DiHETE and 18‐HEPE increased in the carriers of the TT genotype but remained unchanged in the carriers of the CC genotype (Figure 5B, Table S11, Supporting Information). Absolute changes of EPA‐derived lipid mediators (15‐,8‐, and 5‐HEPE and 14,15‐ and 17,18‐DiHETE) were positively correlated with the changes of EPA concentrations in the PL fraction (Figure S4B, Supporting Information). Concentrations of DHA‐derived LOX metabolites 8‐hydroxydocosahexaenoic acid (HDoHE), 17‐HDoHE, 4‐HDoHE, and CYP metabolites 19,20‐epoxydocosapentaenoic acid (EpDPE) and 16,17‐EpDPE decreased significantly in the carriers of the CC genotype but remained unchanged in TT genotype (*p*_unadjusted_ < 0.05 for genotype–diet interaction for all) (Figure 5C, Table S11, Supporting Information). LA derived 9(10)‐EpOME, 9,10‐ and 12,13‐DiHOME, 9,10,11‐TriHOME and 9,12,13‐TriHOME (12a/b) increased and EKODE and 12(13)‐EpOME decreased in the carriers of the TT genotype, but remained unchanged in the carriers of the CC genotype (Figure 4C). DGLA derived lipid mediators remained unchanged in the carriers of the TT genotype, but 8‐HETrE significantly and 15‐HETrE nominally (*p*_unadjusted_ = 0.03) decreased in the carriers of the CC genotype (Figure 4B). Interestingly, concentrations of AA derived 5,6‐, 11,12‐ and 14‐15‐DiHETrE, 5‐ and 8‐HETE decreased in the carriers of the CC genotype, whereas only 8,9‐ and 11,12‐DiHETrE decreased in the carriers of the TT genotype in response to high‐ALA diet.

## 4 Discussion

In this genotype‐based randomized controlled trial we showed that the *FADS1* rs174550 genotype impacts metabolism of LC‐PUFAs in response to high‐LA and high‐ALA diets. Our findings show that in subjects with genetically high D5D enzyme activity, carriers of the *FADS1* rs174550‐TT genotype, the concentration of EPA‐ and EPA‐derived lipid mediators increased in response to high‐ALA diet. On contrary, there was no increase in AA or AA‐derived lipid mediator concentrations in the carriers of TT genotype in response high‐LA diet.

In response to the high‐ALA diet plasma EPA concentrations increased in the carriers of the *FADS1* rs174550‐TT genotype, but not in the carriers of the CC genotype reflecting the expected greater D5D activity in the TT genotype. Our results provide support to a study by Gillingham et al.^[^ ^32^ ^]^ where the carriers of *FADS1* and *FADS2* major alleles had higher increase in EPA in response to a high‐ALA diet compared to the minor allele carriers. On this note, increased dietary stearidonic acid (SDA) increases EPA concentrations in erythrocyte membranes more efficiently than ALA alone,^[^ ^33^, ^34^ ^]^ highlighting also D6D mediated conversion of ALA to SDA and further to EPA as an additional important step in the pathway.

In response to the high‐LA diet D5D index and concentrations of AA remained unchanged in the carriers of the TT genotype but decreased in the carriers of the CC genotype. The first step of n‐6 PUFA metabolism, D6D mediated desaturation of LA to GLA, is suggested to be the rate limiting step in PUFA metabolism.^[^ ^35^ ^]^ Botanical oil rich in GLA (Borage oil), but not the one rich in LA (soybean oil), is shown to modestly increases the proportion of AA in serum total lipids independently of the *FADS1* rs174537 genotype.^[^ ^36^ ^]^ However, in their study the daily LA dose from soybean oil was ≈5 g day^−1^ on top of the daily intake and did not alter concentrations of any long chain n‐6 PUFAs. In our study the total dietary intake of LA increased from ≈9 to 29 g day^−1^ and significantly increased LA concentration in plasma lipids, but the concentration of GLA and D6D index remained unchanged.

One possibility for the observed differences in the AA and EPA changes in the high‐LA and high‐ALA diets, respectively, is that the enzymatic pathway for LA, but not for ALA, is saturated by the high levels of substrate LA. Changes in dietary LA have only minor effects on plasma/serum phospholipid AA proportions in subjects consuming typical Western diet with high LA intake.^[^ ^37^ ^]^ However, supplementation with GLA and AA increases AA in plasma/serum in the general population.^[^ ^37^ ^]^ Furthermore, proportions of plasma AA in vegans (with reduced dietary intake of AA, EPA, DPA, and DHA) are not lower compared to omnivores whereas EPA and DHA are.^[^ ^38^ ^]^ It is therefore possible that under a diet with relatively high LA intake compared to ALA intake, the desaturase and elongase mediated metabolism of LA to AA reaches its maximum physiological rates. D5D and D6D have higher maximal activity for n‐3 than n‐6 PUFAs.^[^ ^39^ ^]^ It is therefore, altogether not surprising that the plasma EPA concentration increased in the carriers of the TT genotype. Dietary AA and DHA reduce *FADS1* and *FADS2* expression in baboon liver^[^ ^40^ ^]^ and AA and EPA reduce *Fads1* and *Fads2* expression on 3T3‐L1 adipocytes.^[^ ^41^ ^]^ It is possible that relatively high intake of n‐3 PUFAs (ALA, EPA, DHA) already at the baseline, based on the 4‐day food records, suppresses conversion of ALA to EPA and affected especially the carriers of the *FADS1* rs174550‐CC genotype. However, the relative contribution of D5D and D6D activity to the total lipid bioavailability remains to be evaluated in further studies.

Dietary sources of high nutritional value, and enriched in ALA, most often also contain significant amounts of LA. By replacing food items rich in LA, e.g., vegetable oils, by oils with greater ALA content in relation to levels of LA, the increase of ALA with decreased or stable LA intake could be achieved. CSO contains 38 mol% ALA and 16 mol% LA of total fatty acids.^[^ ^42^ ^]^ At the baseline, the participants’ diet contained almost four times more LA compared to ALA, as it is typical in Finland.^[^ ^43^ ^]^ In the current study, supplementation with CSO increased dietary intakes of both ALA and LA, and the ratio of dietary LA to ALA decreased from ≈4:1 to ≈1:1 in the high‐ALA diet. Increase in tissue EPA is achieved by decreasing LA intake while maintaining constant intake of ALA.^[^ ^44^, ^45^ ^]^

Free plasma circulatory concentrations of ALA‐derived hydroxy‐, epoxy‐, and dihydroxy‐PUFAs significantly increased in response to the 8‐week high‐ALA diet. Specifically, EPA‐derived hydroxy‐ and dihydroxy PUFAs increased in the carriers of the TT genotype, although the nominally significant genotype–diet interaction was observed only for 15‐HEPE. Proportions and concentrations of DHA decreased in both genotypes but interestingly, DHA‐derived hydroxy‐, and epoxy PUFAs decreased only in the carriers of the CC genotype. Furthermore, we could show that absolute changes of ALA and EPA concentrations in plasma phospholipids and lipid mediators derived from phospholipids metabolism are positively correlated. These findings regarding n‐3 PUFA‐derived lipid mediators are in line with our previous dietary intervention trial conducted with CSO and fatty fish, i.e., rich in EPA+DHA,^[^ ^46^ ^]^ in which elevated intake of dietary ALA and EPA+DHA, led to increasing concentrations of free circulatory ALA‐ and EPA/DHA‐derived lipid mediators, as well as phospholipid conjugated ALA and EPA+DHA in plasma. High erythrocyte membrane EPA proportions associate with high serum concentrations of EPA‐derived hydroxy‐, epoxy‐, and dihydroxy‐FAs^[^ ^47^ ^]^ and Greupner et al.^[^ ^48^ ^]^ reported increased concentrations of ALA‐ and EPA‐derived lipid mediators in response to dietary ALA (linseed oil, ALA 14 g day^−1^, 12 weeks) in male subjects with low basal EPA+DHA status. Our observed increase in EPA‐derived hydroxy‐ and dihydroxy‐PUFAs in the carriers of the TT genotype in the high‐ALA diet is similar, although with lower magnitude than in response to EPA (1.56 g day^−1^) and DHA (1.14 g day^−1^) supplementation in men in a 12‐week trial.^[^ ^49^ ^]^ Taken together, our results show that gene × diet factors are important determinants of metabolic health status wherein dietary PUFAs^[^ ^18^ ^]^ and lipid mediators originating from these are known modulators of the pathophysiology associated with T2D and cardiovascular diseases.^[^ ^11^, ^13^ ^]^

Our data indicate differences on how plasma concentrations of n‐6‐ and n‐3‐derived lipid mediators are altered in response to dietary changes. Changes in precursor n‐3 PUFA status in response to the high‐ALA diet were reflected by altered concentrations of circulating ALA‐ and EPA‐derived lipid mediators. Surprisingly, in the high‐LA diet the changes in LA‐derived lipid mediators were minor. In our previous FADSDIET1 study we reported an increase in the majority of the LA‐derived lipid mediators in response to a 4‐week high‐LA diet.^[^ ^5^ ^]^ Further studies are needed to reveal whether the longer duration of high‐LA diet modifies LA metabolism into its lipid mediators.

Overall, changes in AA‐derived lipid mediators were minor compared to n‐3 PUFA‐derived lipid mediators. CYP enzymes convert AA to epoxides (EpETrEs) which are further metabolized to dihydroxy fatty acids (DiHETrEs) by soluble epoxide hydrolase. Of interest, concentrations of 5,6‐, 8,9‐, 11,12‐, and 14,15‐DiHETrEs decreased significantly in the carriers of the *FADS1* rs174550‐CC genotype, following high‐LA diet and to a some extent in high‐ALA, but the concentrations of the corresponding EpETrEs remained unchanged. This decrease in DiHETrEs may be explained by the significant reduction in PL and CE conjugated AA in the carriers of the CC genotype in the high‐LA diet and in both genotypes in the high‐ALA diet. Also, the tendency towards stronger decrease in the concentrations of AA derived lipid mediators in the carriers of the CC genotype compared to TT genotype in a high‐ALA diet could be partially explained by the competition of AA (decreased in both genotypes) and EPA (which increased only in the carriers of the TT genotype) on the enzymes producing lipid mediators. Altogether, this suggest that changes in concentrations of n‐3 PUFA‐derived lipid mediators are more easily achieved by high‐ALA diet with increasing magnitude of change in the carriers of the rs174550‐TT genotype, than in n‐6 PUFA‐derived lipid mediators using a high‐LA diet.

A randomized controlled design and a good compliance of the participants are two the major strengths of our study. Participants were all aging male and results are not generalizable for a broader aged population. Power calculations were based on the change of AA proportion in the CE fraction in response to the high‐LA diet in the FADSDIET1 trial.^[^ ^5^ ^]^ Since power calculations were based on fatty acids, also nominally significant (*p*_unadjusted_ < 0.05) results were considered for lipid mediators. Although the unbalanced high‐ALA arm (CC, *n* = 21 and TT, *n* = 39) is a limitation, analysis of randomly selected high‐ALA subjects (CC, *n* = 21 vs TT, *n* = 21) replicated the same results for changes in EPA and EPA‐ and DHA‐derived lipid mediators as for the complete group analysis. D5D and D6D indices were estimated by the fatty acid ratios and the gene or protein expression of *FADS1* or *FADS1* activity was not measured. This is considered as a limitation of the study since the estimation of D6D activity is based on the LA concentration which was increased in response to high‐LA diet.

Our results show that both concentrations of plasma PUFAs and lipid mediators are regulated by the interactions between *FADS1* rs174550 genotype and dietary PUFAs. Findings regarding the observed *FADS1* rs174550 genotype–diet interaction in plasma fatty acids should be considered when conducting future trials with PUFA supplementation. Furthermore, the main results from genotype–diet interaction data presented in this study may help tailor dietary regimens individually. This will allow optimization of disease prevention through dietary modifications, since circulating PUFAs strongly associate with the risk of T2D and cardiovascular diseases.^[^ ^14^, ^15^, ^16^, ^17^ ^]^

## Conflict of Interest

The authors declare no conflict of interest.

## Author Contributions

Conceptualization: M.A.L./V.D.d.M./K.A.V./M.L./J.P./U.S. Funding: M.A.L./M.L./C.E.W./U.S. Methodology: J.K./J.Å./C.E.W. Investigation: T.M./M.A.L./J.K./V.D.d.M./T.S./K.A.V./U.S. Supervision: M.A.L./U.S. Formal analysis: T.M./M.A.L./J.K. Visualization: T.M./J.K. Writing: T.M./M.A.L./J.K./V.D.d.M./T.S./K.A.V./C.E.W./J.P./U.S.

## Supporting information

supplementary information

supplementary information

supplementary information

supplementary information

Supplementary information

## Data Availability

The data are not publicly available due to privacy or ethical restrictions.
