# Supplementary figures and images for: The FADS1 rs174550 Genotype Modifies the n‐3 and n‐6 PUFA and Lipid Mediator Responses to a High Alpha‐Linolenic Acid and High Linoleic Acid Diets
Source: Mol Nutr Food Res. 2022 Nov 11;66(24):2200351. doi: 10.1002/mnfr.202200351 (PMC10077898; doi:10.1002/mnfr.202200351)

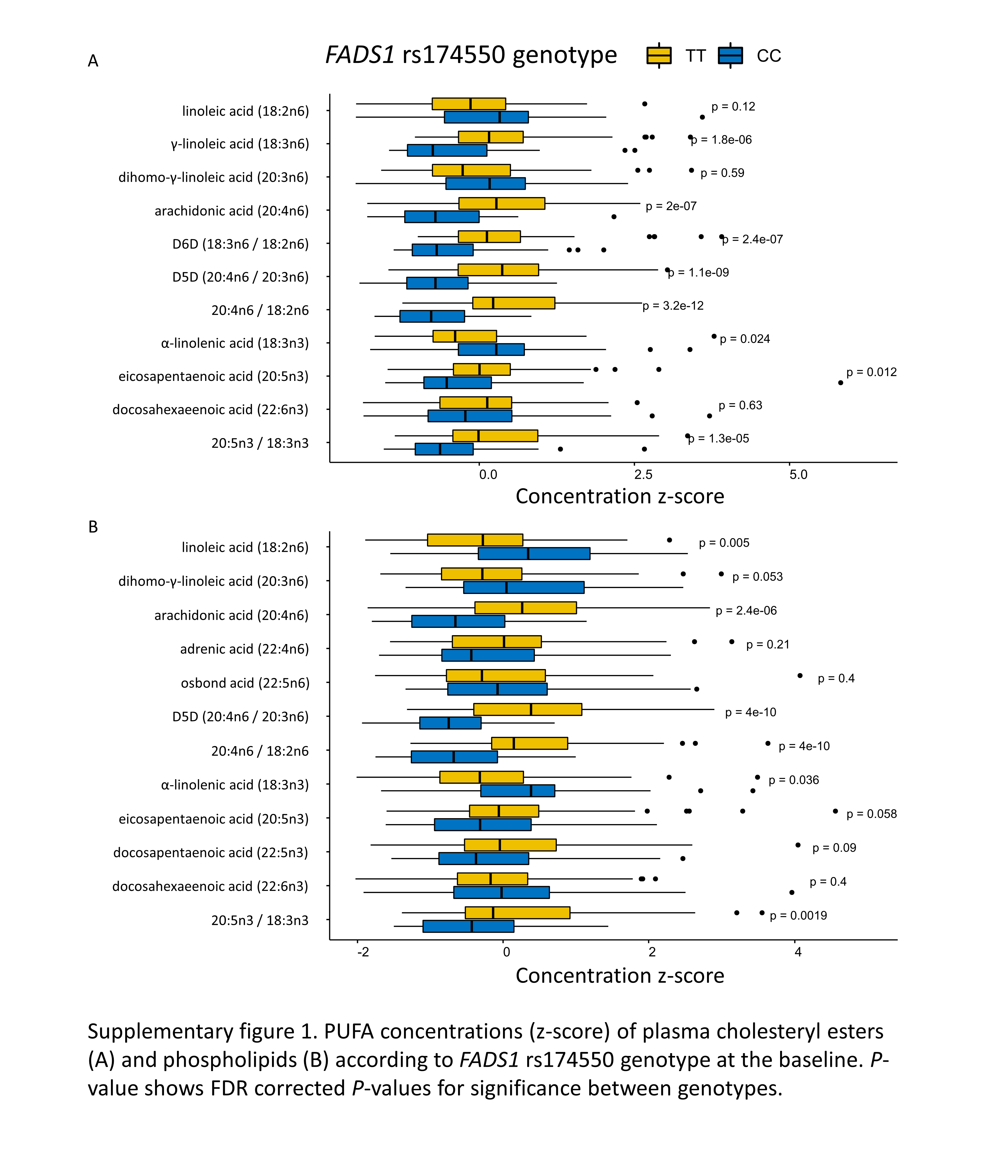

Supplement: Supplementary file 1 — supplementary information [file MNFR-66-0-s005.tif]

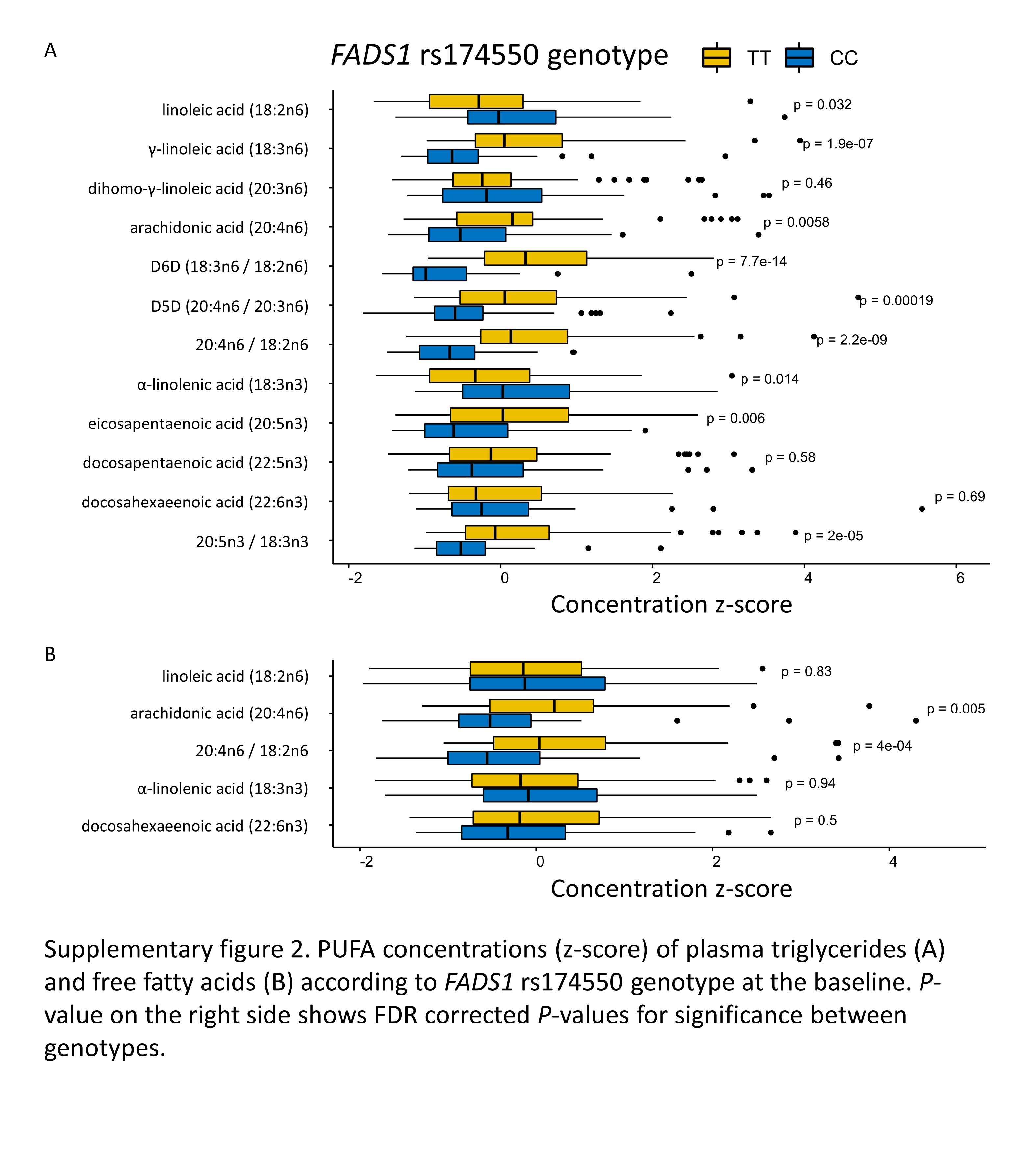

Supplement: Supplementary file 2 — supplementary information [file MNFR-66-0-s003.tif]

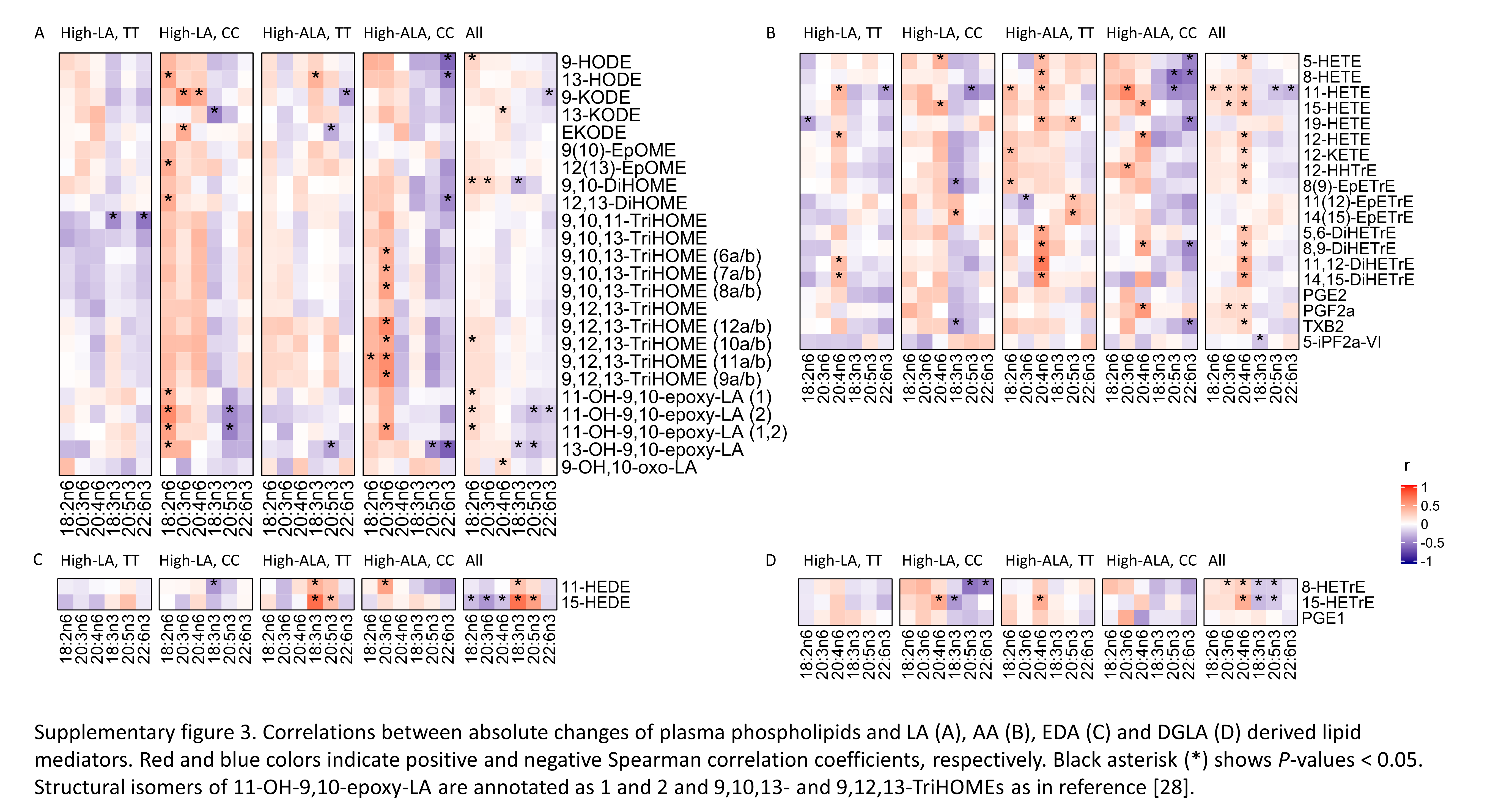

Supplement: Supplementary file 3 — supplementary information [file MNFR-66-0-s004.tif]

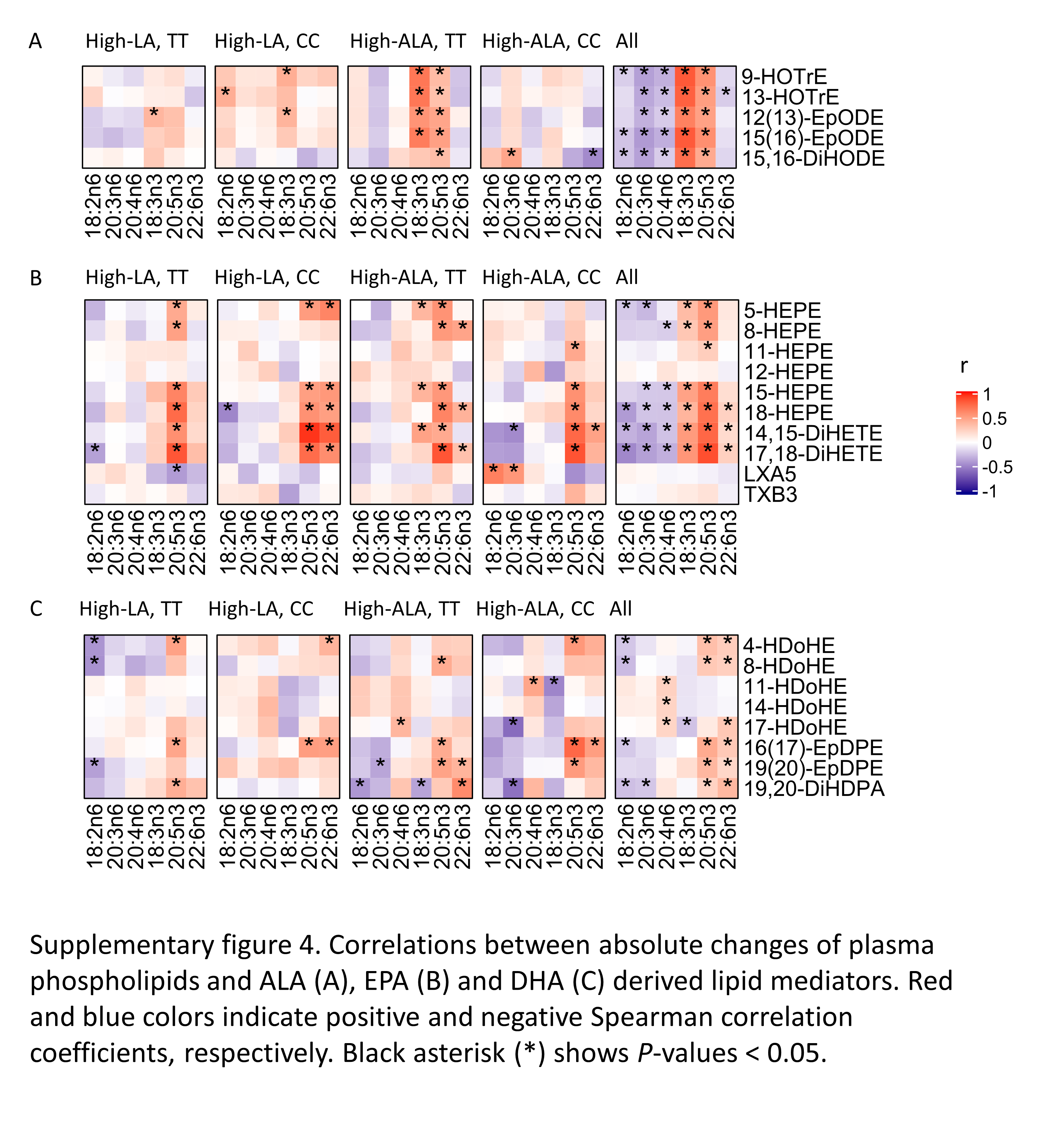

Supplement: Supplementary file 4 — supplementary information [file MNFR-66-0-s002.tif]
